# Optimization of Ammonium Sulfate Concentration for Purification of Colorectal Cancer Vaccine Candidate Recombinant Protein GA733-FcK Isolated from Plants

**DOI:** 10.3389/fpls.2015.01040

**Published:** 2015-11-27

**Authors:** Se-Ra Park, Chae-Yeon Lim, Deuk-Su Kim, Kisung Ko

**Affiliations:** Therapeutic Protein Engineering Lab, Department of Medicine, College of Medicine, Chung-Ang UniversitySeoul, South Korea

**Keywords:** ammonium sulfate, colorectal cancer, GA733-Fc, recombinant vaccine, transgenic plant

## Abstract

A protein purification procedure is required to obtain high-value recombinant injectable vaccine proteins produced in plants as a bioreactor. However, existing purification procedures for plant-derived recombinant proteins are often not optimized and are inefficient, with low recovery rates. In our previous study, we used 25–30% ammonium sulfate to precipitate total soluble proteins (TSPs) in purification process for recombinant proteins from plant leaf biomass which has not been optimized. Thus, the objective in this study is to optimize the conditions for plant-derived protein purification procedures. Various ammonium sulfate concentrations (15–80%) were compared to determine their effects on TSPs yield. With 50% ammonium sulfate, the yield of precipitated TSP was the highest, and that of the plant-derived colorectal cancer-specific surface glycoprotein GA733 fused to the Fc fragment of human IgG tagged with endoplasmic reticulum retention signal KDEL (GA733^P^-FcK) protein significantly increased 1.8-fold. SDS-PAGE analysis showed that the purity of GA733^P^-FcK protein band appeared to be similar to that of an equal dose of mammalian-derived GA733-Fc (GA733^M^-Fc). The binding activity of purified GA733^P^-FcK to anti-GA733 mAb was as efficient as the native GA733^M^-Fc. Thus, the purification process was effectively optimized for obtaining a high yield of plant-derived antigenic protein with good quality. In conclusion, the purification recovery rate of large quantities of recombinant protein from plant expression systems can be enhanced via optimization of ammonium sulfate concentration during downstream processes, thereby offering a promising solution for production of recombinant GA733-Fc protein in plants.

## Introduction

Mammalian, yeast, and insect cell cultures are used to produce recombinant subunit vaccines owing to their ability to express proteins in a manner similar to that observed in the native organism (Ko, 2014). However, expensive culture media and purification steps required for recovering recombinant proteins expressed in the cells of these organisms increase the cost of recombinant subunit vaccine production. In addition, most subunit vaccines produced in these systems are heat sensitive and require parenteral delivery, which restricts the use of recombinant subunit vaccines in many developing countries where health systems are not well equipped (Rigano and Walmsley, 2005). For use in plant molecular biofarming, the tobacco plant has several advantages over other plants, such as highly efficient transformation and regeneration, relatively short period for biomass production, and easy homogenization processing for protein purification due to soft leaf tissue (Jamal et al., 2009, 2012; Song et al., 2015). The tumor-associated antigen GA733, a glycoprotein that is highly expressed on the cell-surface of colorectal carcinomas, has previously been fused with the human immunoglobulin Fc fragment carrying the ER retention sequence, to produce the recombinant antigen-antibody complex GA733-FcK in a plant expression system (Lu et al., 2012; Park et al., 2015a,b). Moreover, expression of the recombinant protein GA733-FcK has been successful in a plant system (Lim et al., 2015). Optimization of the protein purification process is essential for the successful production of the tagged recombinant therapeutic protein in the total soluble protein (TSP) recovery step during the downstream process (Geyer et al., 2005; Aspelund and Glatz, 2010; Kim et al., 2015). To purify the tagged recombinant proteins, the TSPs must be extracted, separated, and eventually isolated, from the plant biomass debris (Desai et al., 2002). In general, 35% ammonium sulfate is often applied for precipitation of TSPs from plant extracts (Lim et al., 2015). However, the ammonium sulfate concentration for the precipitation of TSPs harboring GA733-FcK has not been optimized. In this study, a recombinant colorectal cancer vaccine candidate fusion protein GA733-FcK expressed in transgenic *Nicotiana tabacum* plants was purified by protein-G affinity chromatography. Thus, we optimized ammonium sulfate TSP precipitation conditions to increase the recovery rate of GA733-FcK in transgenic plant leaves.

## Materials and Methods

### Plant Material

**FIGURE 1:**
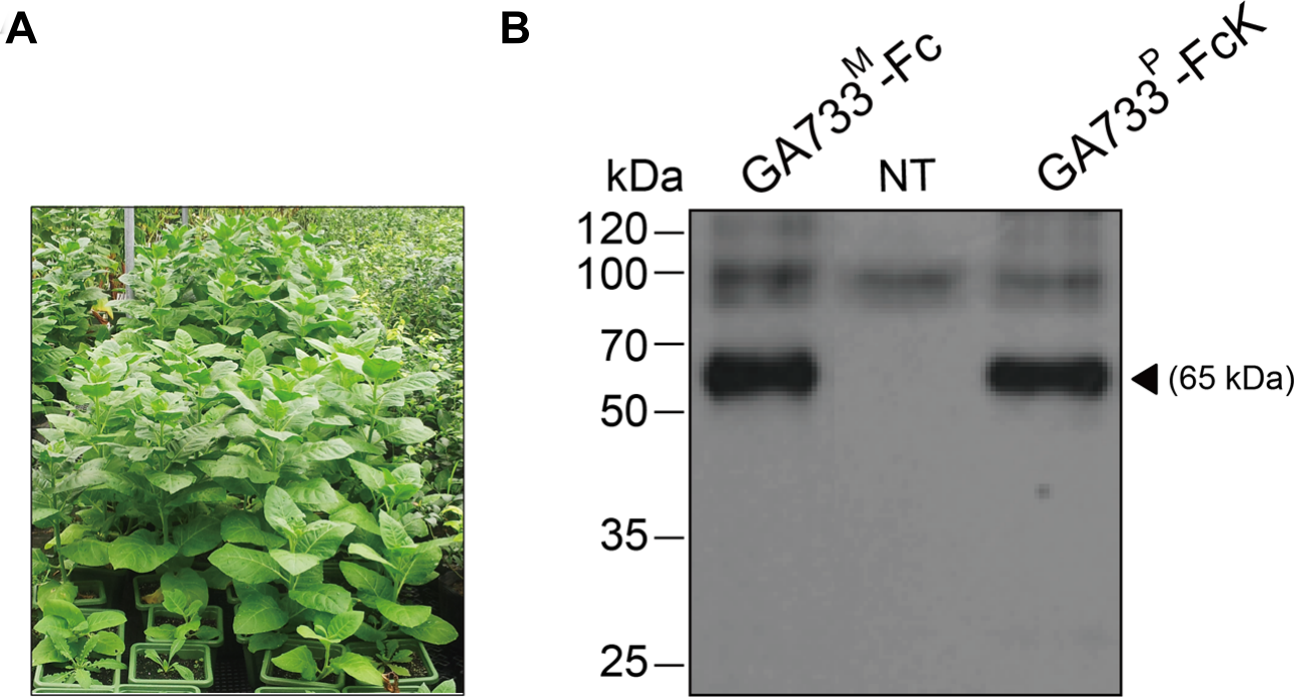
**Growth of transgenic plants expressing GA733-FcK (A) in a greenhouse and immunoblot analysis of the plant-derived GA733-FcK (GA733^P^-FcK) and mammalian-derived GA733-Fc (GA733^M^-Fc) (B).** Plant leaf samples were homogenized in 1 × PBS, separated by 10% SDS-PAGE, and transferred onto nitrocellulose membrane. Lane 1, positive control; GA733^M^-Fc (68 kDa); lane 2, non-transgenic plant (NT); and lane 3, GA733^P^-FcK. The bands were detected by anti-human Fc IgG conjugated to HRP.

Seedlings of transgenic tobacco (*N. tabacum*) plants expressing the recombinant protein GA733-FcK (Lu et al., 2012) were transplanted into a pot containing soil and grown in a greenhouse (**Figure 1A**).

### Removal of Chloroplasts

**FIGURE 2:**
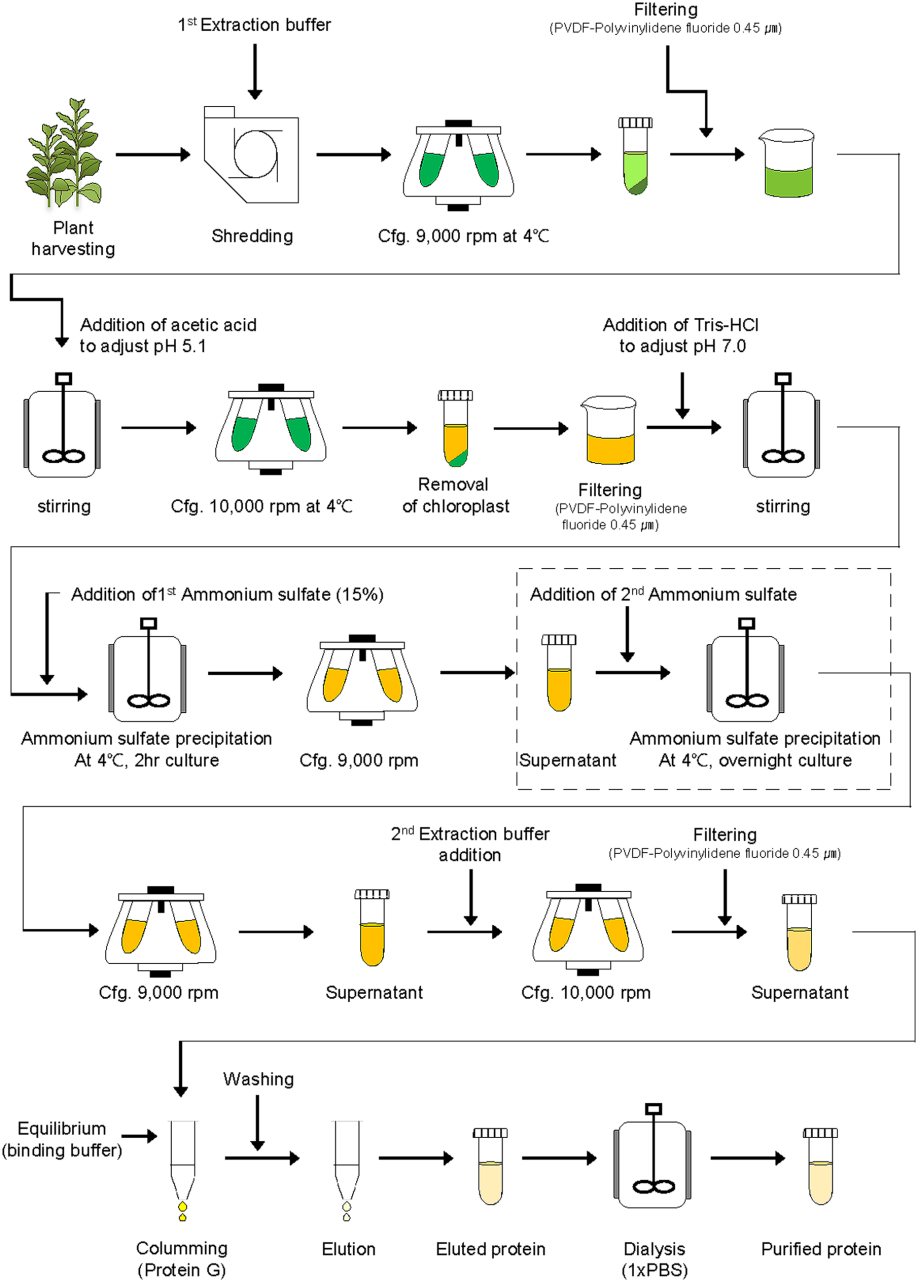
**Schematic diagram of downstream processing of recombinant protein GA733-FcK from plant leaf biomass.** The second ammonium sulfate application (15, 30, 35, 40, 50, 60, 70, and 80%) was performed for TSP precipitation (the dotted lined rectangular box).

For purification of the plant-derived recombinant protein GA733-FcK (GA733^P^-FcK), tobacco plant leaves were homogenized in an HR2094 blender (Philips, Seoul, Korea) using extraction buffer (37.5 mM Tris-HCl, pH 7.5; 50 mM NaCl; 15 mM EDTA; 75 mM sodium citrate; and 0.2% sodium thiosulfate) (**Figure 2**). After centrifugation at 9,000 × *g* for 30 min at 4°C, the supernatant was filtered through a Miracloth (Biosciences, La Jolla, CA, USA), and pH of the filtered solution reduced to 5.1 by adding 99.0% ultrapure acetic acid, pH 2.4. The solution was again centrifuged at 10,000 × *g* for 30 min at 4°C.

### Protein Precipitation

After removal of the chloroplasts, the pH of the solution was brought up to 7.0 by adding 3 M Tris-HCl, and ammonium sulfate was added to 15% saturation at 4°C (**Figure 2**). After centrifugation at 9,000 × *g* for 30 min at 4°C, the supernatant was collected. Different concentrations (15, 20, 30, 40, 50, 60, 70, and 80%) of ammonium sulfate were added to the supernatant at 4°C. After incubation at 4°C overnight, the solution was centrifuged again at 9,000 × *g*. The pellet was resuspended in extraction buffer (1/10th of the original solution volume), and the final solution was then centrifuged at 10,000 × *g* for 30 min at 4°C. The supernatant of the extracted sample was then filtered through a 0.45 μm filter (Merck Millipore, Darmstadt, Germany).

### Protein Elution

The TSP solution was loaded onto a 1 ml HiTrap protein G affinity column (Pharmacia, Uppsala, Sweden), and the column was washed with 10 mL binding buffer (0.2 M sodium phosphate, pH 7.0). After washing the column, the protein was eluted with elution buffer (1 M glycin-HCl, pH 2.7). One-milliliter aliquots of the fraction were collected in microtubes containing 55 μL of neutralizing buffer (1 M Tris-HCl, pH 9.0).

### Dialysis

The eluted samples of GA733^P^-FcK were dialyzed against 1 × PBS buffer (137 mM NaCl, 10 mM Na_2_HPO_4_, 2.7 mM KCl, and 2 mM KH_2_PO_4_) twice at 4°C for 1 h 30 min, and the final dialysis was performed overnight at 4°C. Dialyzed samples were frozen in liquid nitrogen and stored at -80°C until further analysis.

### SDS-PAGE

Fresh harvested leaf samples (100 mg) were ground in 300 μL of 1 × PBS buffer (137 mM NaCl, 10 mM Na_2_HPO_4_, 2.7 mM KCl, and 2 mM KH_2_PO_4_). A 20 μL sample was mixed with 4 μL loading buffer (1 M Tris-HCl, 50% glycerol, 10% SDS, 5% 2-mer captoethanol, and 0.1% bromophenol blue) and loaded onto the 12% protein gel. The proteins were electrophoresed using 1 × SDS running buffer (25 mM Tris-HCl, 200 mM glycine, 0.1% [w/v] SDS). The protein gel was stained with Coomassie blue staining solution (10% acetic acid [v/v], 30% methanol [v/v], 0.01% Coomassie blue [w/v]) by shaking at room temperature (RT) for 30 min. The gel was de-stained with 10% acetic acid by shaking at RT.

### Immunoblot Analysis

The proteins electrophoresed through the gel were transferred to a nitrocellulose membrane (Millipore Corp., Billerica, MA, USA). Membranes were blocked with 5% skim milk powder (Sigma, St. Louis, MO, USA) in 1 × PBS-T buffer (1 × PBS plus 0.5% [v/v] Tween 20) at RT for 2 h. The membrane was incubated for 1 h 30 min at RT with goat anti-human Fcγ (1:15,000) recognizing the human Fc fragment portion of GA733-FcK. The protein bands were detected using SuperSignal chemiluminescence substrate (Pierce, Rockford, IL, USA). Protein bands were visualized by exposing the membrane to an X-ray film (Fuji, Tokyo, Japan) using a chemiluminescence substrate (Pierce).

### Surface Plasmon Resonance (SPR)

Steady-state equilibrium binding of GA733^P^-FcK and GA733^M^-Fc were analyzed at 25°C using a ProteOn XPR36 surface plasmon resonance (SPR) biosensor (Bio-Rad Labs, Hercules, CA, USA) (Khurana et al., 2009; Kim et al., 2015). Anti-GA733 mAb was injected for immobilization on the chip in the horizontal orientation of the ProteOn XPR36 fluidics at a flow rate of 40 μL/min for 90 s (60 μL). GA733^P^-FcK purified from plants (1 and 2 μg) and GA733^M^-Fc (1 and 2 μg) were injected in the vertical orientation of the ProteOn XPR36 fluidics for 6 min (150 μL) at 25 μL/min, allowing them to be captured by anti-GA733 mAbs immobilized on the chip. The 1 × SDS running buffer was injected simultaneously in the sixth channel to correct for loss of the captured supernatant GA733^P^-FcK or GA733^M^-Fc from the chip sensor surface during the experiment, as described by Nahshol et al. (2008). The data for binding kinetics of the anti-GA733 mAbs to both GA733^P^-FcK and GA733^M^-Fc were analyzed using Bio-Rad ProteON manager software. Affinity measurements were calculated using the Langmuir with Mass Transfer Algorithm (Khurana et al., 2009).

## Results

### Expression of Recombinant GA733^P^-FcK Protein in Transgenic Plants

The seedlings of transgenic plants expressing GA733-FcK (Lu et al., 2012) were transplanted into pots containing soil and grown in a greenhouse (**Figure 1A**). Western blot analysis with anti-human Fcγ antibody was conducted to confirm the expression of GA733^P^-FcK in the seedling leaf. GA733-FcK protein band was detected at approximately 65 kDa, similar to the band observed for GA733^M^-FcK (positive control) (**Figure 1B**). No band was observed in the non-transgenic plant (NT).

### Effect of the Second Ammonium Sulfate Concentration on TSP Precipitation

**FIGURE 3:**
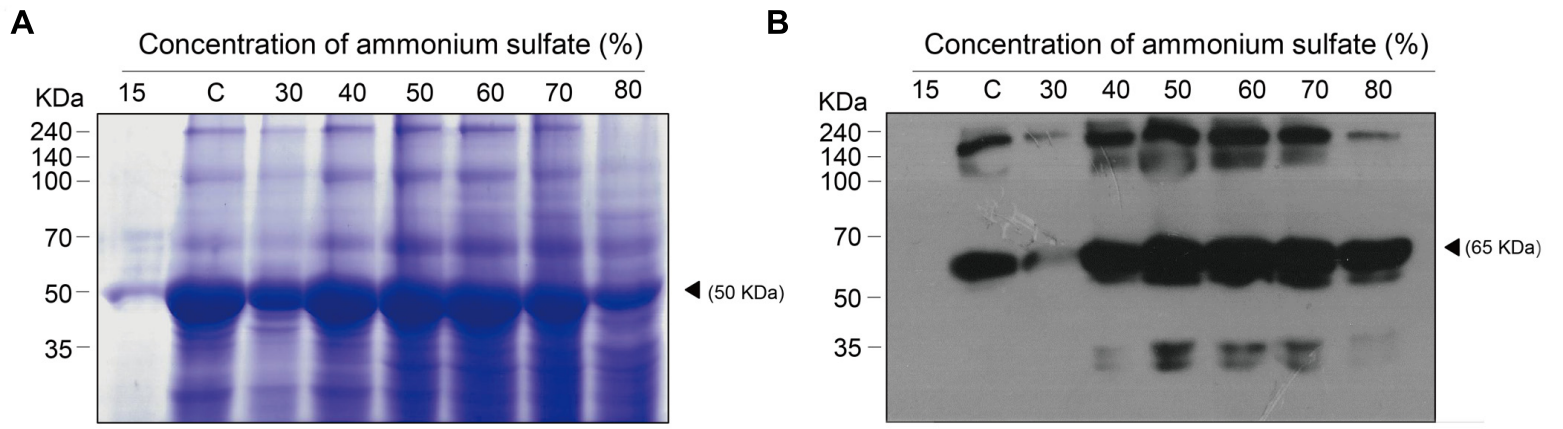
**SDS-PAGE to analyze the levels of TSPs precipitated from biomass extracts of transgenic plants expressing recombinant GA733-FcK (A) and western blot analysis to specifically detect recombinant GA733^P^-FcK in the precipitated TSPs (B).** **(A)** TSPs were visualized on a Coomassie-stained SDS-PAGE gel. **(B)** GA733^P^-FcK proteins were detected by anti-human Fc IgG conjugated to HRP. White and solid arrowheads indicate RuBisCO and GA733-FcK, respectively. C; 35% ammonium sulfate used as a control.

Total soluble proteins isolated from transgenic plant leaf biomass and GA733^P^-FcK present therein were analyzed by SDS-PAGE and western blot analyses, respectively (**Figures 3A,B**, respectively). In order to confirm the effect of the second ammonium sulfate concentration for TSP precipitation after homogenization of leaf biomass and removal of chloroplasts, the TSPs were precipitated using various concentrations (15–80%) of the second ammonium sulfate (**Figure 2**, rectangle with a dotted line). TSP precipitation in the plant leaf extraction solutions was visualized on a Coomassie-stained gel. The levels of precipitated TSP in the extracts were the highest with 40–60% of ammonium sulfate (**Figure 3A**). At 15%, the density of TSP bands was the weakest, followed by those at 80 and 30%. The GA733^P^-FcK protein band in the TSP fraction was detected at approximately 65 kDa by the secondary antibody anti-human Fc IgG conjugated to HRP at 30–80% ammonium sulfate, whereas no protein band was detected at 15% (**Figure 3B**). Among all the tested concentrations of ammonium sulfate, the density of GA733^P^-FcK protein bands was stronger at 50, 60, and 70% ammonium sulfate than that at other concentrations (15, 30, 40, and 80%) (**Figure 3B**).

### Effect of the Second Ammonium Sulfate Concentration on the Purified Recombinant Protein Yield

**FIGURE 4:**
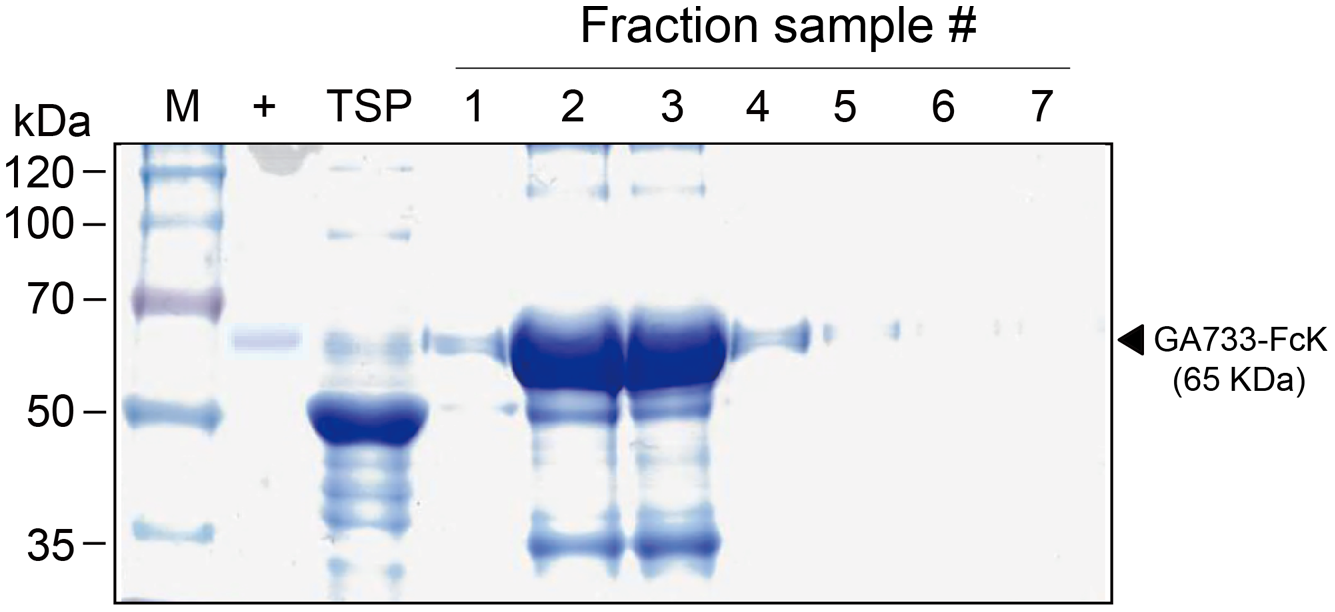
**SDS-PAGE to detect GA733^P^-FcK from eluted fraction samples.** M: protein marker; +: positive control, mammalian-derived GA733-Fc (GA733^M^-Fc); TSP, total soluble protein; 1–7, fraction sample numbers.

**FIGURE 5:**
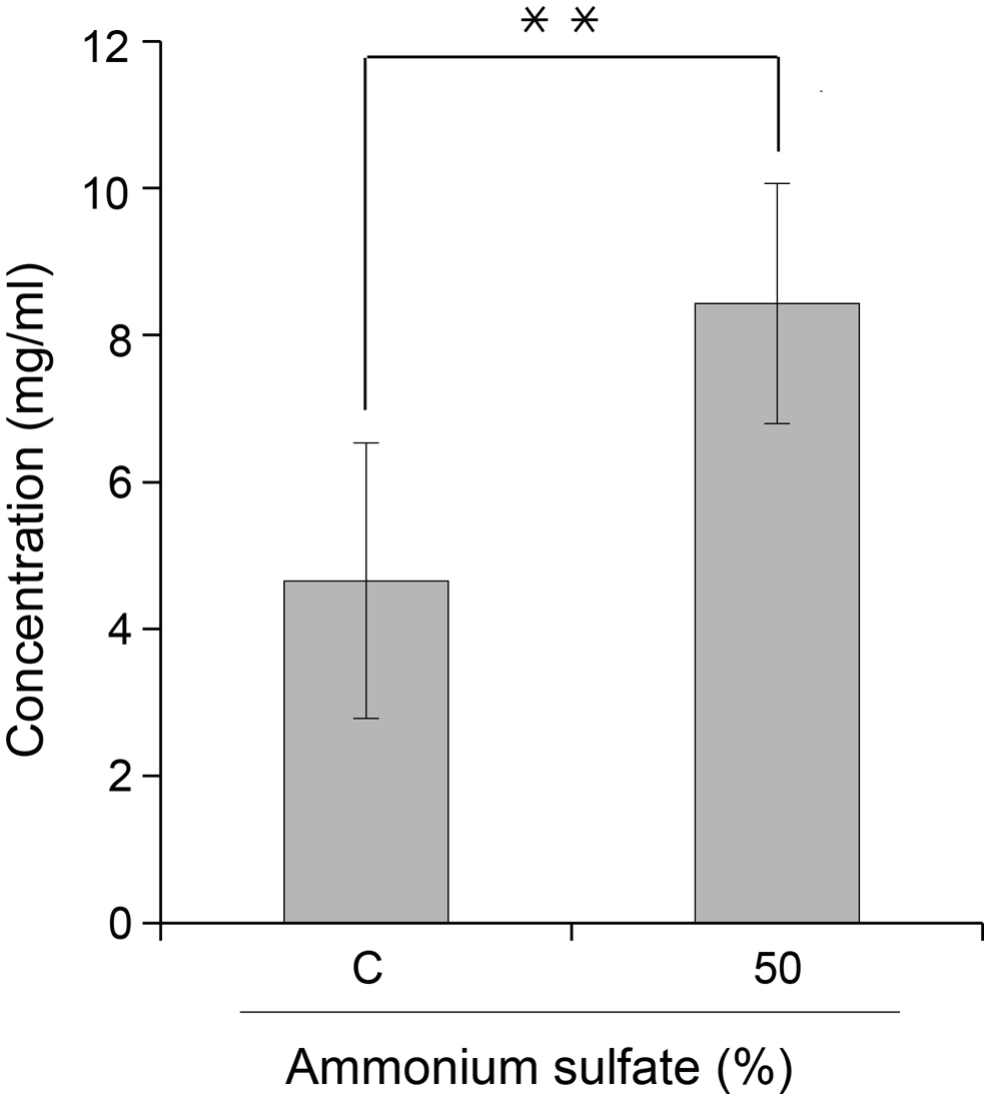
**Effect of ammonium sulfate concentration (%) on purified GA733-FcK yield from transgenic plant leaf biomass.** Comparison of GA733^P^-FcK yield from TSPs treated with 35% (control) and 50% ammonium sulfate. Data represent means and standard errors (^∗∗^*P* < 0.05).

**FIGURE 6:**
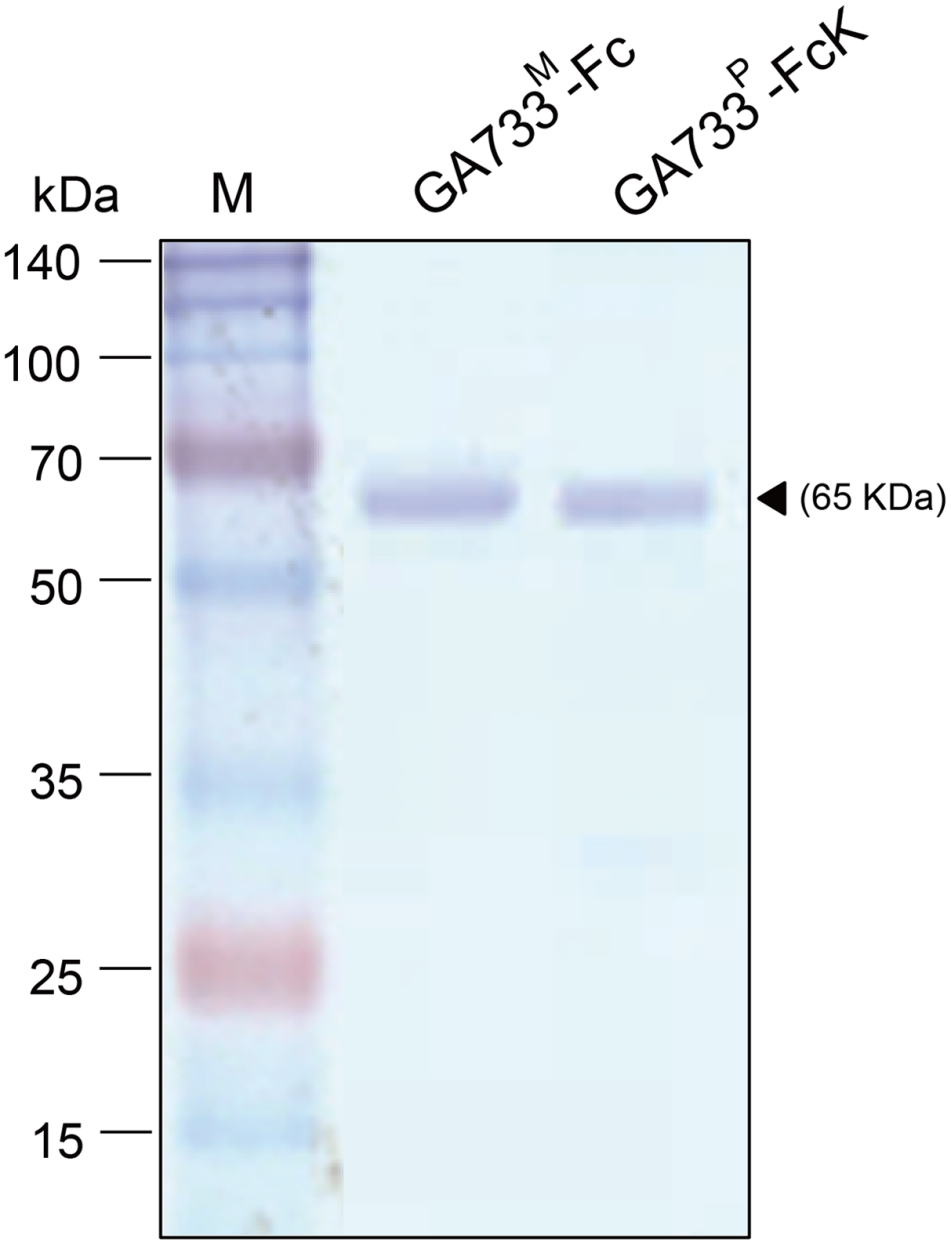
**SDS-PAGE analysis to compare the purity of GA733^P^-FcK with GA733^M^-Fc after optimization of ammonium sulfate concentration (50%).** Black arrowhead indicates a 65 kDa GA733-Fc protein band.

GA733^P^-FcK purification was conducted using protein-G method. We confirmed and optimized the second ammonium sulfate concentration for a downstream procedure as above. Protein fraction samples after elution through protein G column were analyzed by Coomassie blue staining of SDS-PAGE (**Figure 4**). Fractions # 2 and 3 showed strong protein band signals (**Figure 4**). The purified GA733^P^-FcK protein yield with different concentrations of the second ammonium sulfate was analyzed using SDS-PAGE and western blot. The purification yields of GA733^P^-FcK were compared between 35% (control) and 50% of ammonium. The comparison results showed that the optimized concentration of the second ammonium sulfate application (50%) showed 1.8-fold higher yield of GA733^P^-FcK compared to the control concentration (35%) (**Figure 5**). The purity of GA733-FcK protein (65 kDa) purified from plants was similar to the recombinant GA733-Fc protein (65 kDa) purified from an animal expression system (GA733^M^-Fc) (**Figure 6**).

### Binding Activity of Purified GA733^P^-FcK to Anti-GA733 mAb

**FIGURE 7:**
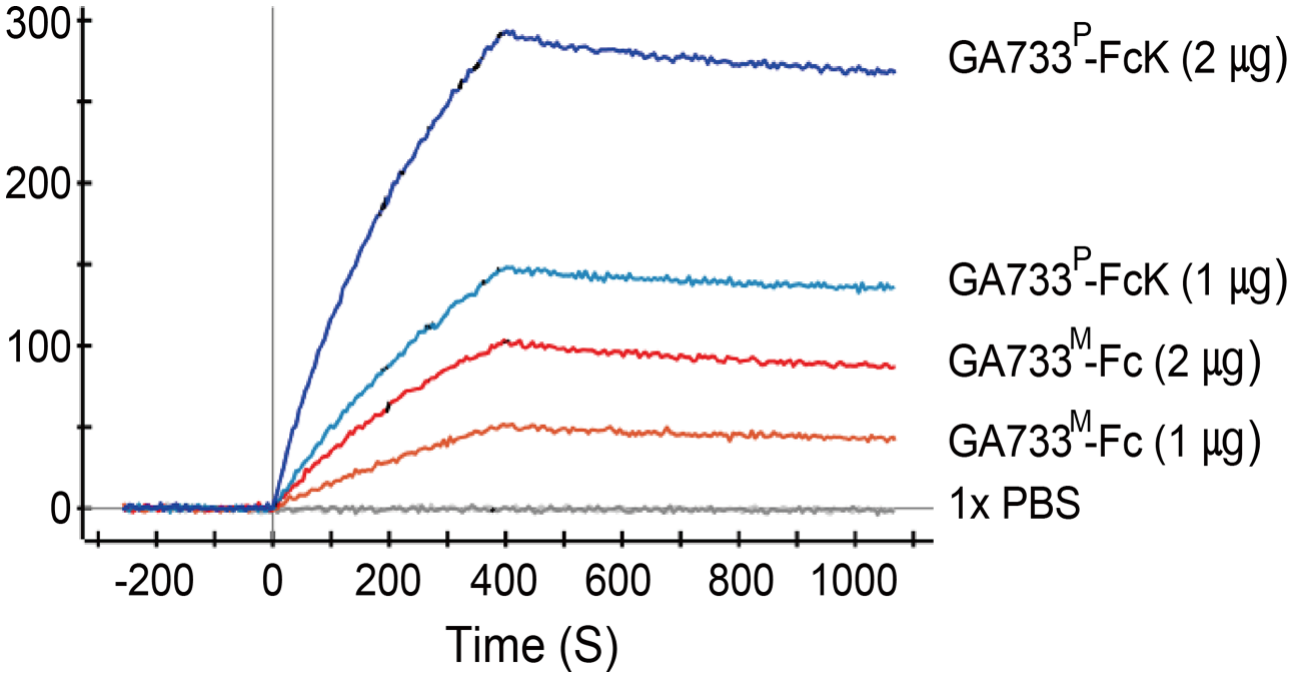
**Surface plasmon resonance interaction analysis of anti-GA733 mAb binding to GA733^P^-FcK and GA733^M^-Fc.** Anti-GA733 mAb was fixed on a GLC-chip. Binding of GA733^P^-FcK to anti-GA733 mAb (mAb CO17-1A) was analyzed using SPR.

Kinetic analysis of binding activity of purified GA733^P^-FcK to anti-GA733 mAb using SPR showed that GA733^P^-FcK had almost three times higher Ru value than parental GA733^M^-FcK. When the amount of GA733^P^-FcK and GA733^M^-Fc applied to the chip was doubled, the Ru values increased almost twice. A solution of 1 × PBS as a negative control did not show any Ru value (**Figure 7**).

## Discussion

Plants used as expression systems for the production of recombinant therapeutic proteins are considered to have several advantages over other expression systems. These include low production cost of biomass containing recombinant proteins. However, the downstream process, including extraction and purification of the recombinant proteins from plant biomass, often is more complicated and expensive than other fermentation systems. Plant tissues contain a wide range of proteins with different properties, and these portions are subjected to extraction and purification during downstream processing. The non-optimized purification steps involved in recovering recombinant proteins expressed in plants increase the production cost of plant-derived proteins. However, existing purification procedures for these recombinant proteins have not been fully optimized (Lu et al., 2012; Lim et al., 2015), and thus, are often inefficient, with low recovery rates (Park et al., 2015a).

The main purpose of this study was to optimize the conditions for precipitation of TSPs with the second ammonium sulfate application during the downstream purification process of the plant-derived GA733-FcK protein. Our data demonstrate the effects of ammonium sulfate concentration on TSP precipitation and the recovery rate of the GA733^P^-FcK from plant biomass. Western blot analysis was conducted to confirm the expression of GA733-FcK in the leaf biomass of transgenic plants grown in a greenhouse. GA733^P^-FcK was detected to be approximately 65 kDa size, similar to the mammalian-derived GA733^M^-Fc (positive control) by the anti-human Fcγ IgG. GA733^P^-FcK yield with different concentrations of the second ammonium sulfate application was analyzed using SDS-PAGE and western blot. We confirmed the level of precipitated TSPs in the extracts using SDS-PAGE. The highest levels of TSPs were observed when 40–60% ammonium sulfate was used. We verified that the TSP bands observed with 80 and 30% ammonium sulfate were weak, followed by that with 15%. The 35% (control) application, which has been applied previously (Lu et al., 2012; Lee et al., 2013; Lim et al., 2015), showed similar band densities as those with 40–60% application. However, western blot analysis showed that the specific band density of GA733^P^-FcK detected by anti-human Fc IgG with 35% application was lower than those detected with 40–60%. These results suggest that the high TSP levels do not always indicate high levels of specific GA733-FcK in the TSPs. Indeed, at 30–80% concentration of ammonium sulfate, GA733-FcK protein band (at ∼65 kDa) was detected, whereas no protein band was detected with 15% ammonium sulfate application. The purification yields (mg/ml) of GA733^P^-FcK were compared between 35% (control) and 50% of ammonium sulfate that resulted in the highest yields for TSPs and GA733-FcK proteins. The comparison results showed that after optimization (using 50% ammonium sulfate), 1.8-fold higher yields were obtained compared to the control (35%). Furthermore, the purity of GA733-FcK purified from plants was similar to that of the recombinant GA733-Fc protein purified from an animal expression system. These results suggest that purification recovery rate of large quantities of recombinant protein from transgenic plant expression systems can be enhanced via optimization of ammonium sulfate concentration during the downstream purification process, thereby offering a promising solution for the production of recombinant GA733-Fc protein in plant expression systems.

## Conflict of Interest Statement

The authors declare that the research was conducted in the absence of any commercial or financial relationships that could be construed as a potential conflict of interest.
